# Supplementary material for: Unveiling charge dynamics of visible light absorbing oxysulfide for efficient overall water splitting
Source: Nat Commun. 2021 Dec 7;12:7055. doi: 10.1038/s41467-021-27199-3 (PMC8651740; doi:10.1038/s41467-021-27199-3)
Supplement: Supplementary file 1 — Supplementary Information [file 41467_2021_27199_MOESM1_ESM.pdf]

Supplementary Information

Unveiling charge dynamics of visible light absorbing oxysulfide for efficient overall water splitting

Vikas Nandal,[*a†] Ryota Shoji,[b†] Hiroyuki Matsuzaki,[*b] Akihiro Furube,[c] Lihua Lin,[d] Takashi Hisatomi,[d] Masanori Kaneko,[e] Koichi Yamashita,[e] Kazunari Domen,[d,f] and Kazuhiko Seki[*a]

[a] Global Zero Emission Research Center, National Institute of Advanced Industrial Science and Technology (AIST), Tsukuba 16-1 Onogawa, Tsukuba, Ibaraki 305-8569, Japan.

[b] Research Institute for Material and Chemical Measurement, National Metrology Institute of Japan (NMIJ), National Institute of Advanced Industrial Science and Technology (AIST), Tsukuba 1-1-1 Higashi, Tsukuba, Ibaraki 305-8565, Japan.

[c] Department of Optical Science, Tokushima University, 2-1 Minamijosanjima-cho, Tokushima 770-8506, Japan.

[d] Research Initiative for Supra-Materials, Interdisciplinary Cluster for Cutting Edge Research, Shinshu University, 4-17-1 Wakasato, Nagano-shi, Nagano 380-8553, Japan.

[e] Elements Strategy Initiative for Catalysts and Batteries (ESICB), Kyoto University, 1-30 Goryo-ohara, Nishikyo-ku, Kyoto 615-8245, Japan.

[f] Office of University Professors, The University of Tokyo, 7-3-1 Hongo, Bunkyo-ku, Tokyo 113-8656, Japan.

*Email: nk.nandal@aist.go.jp, hiroyuki-matsuzaki@aist.go.jp, k-seki@aist.go.jp

† Authors with equal contribution.

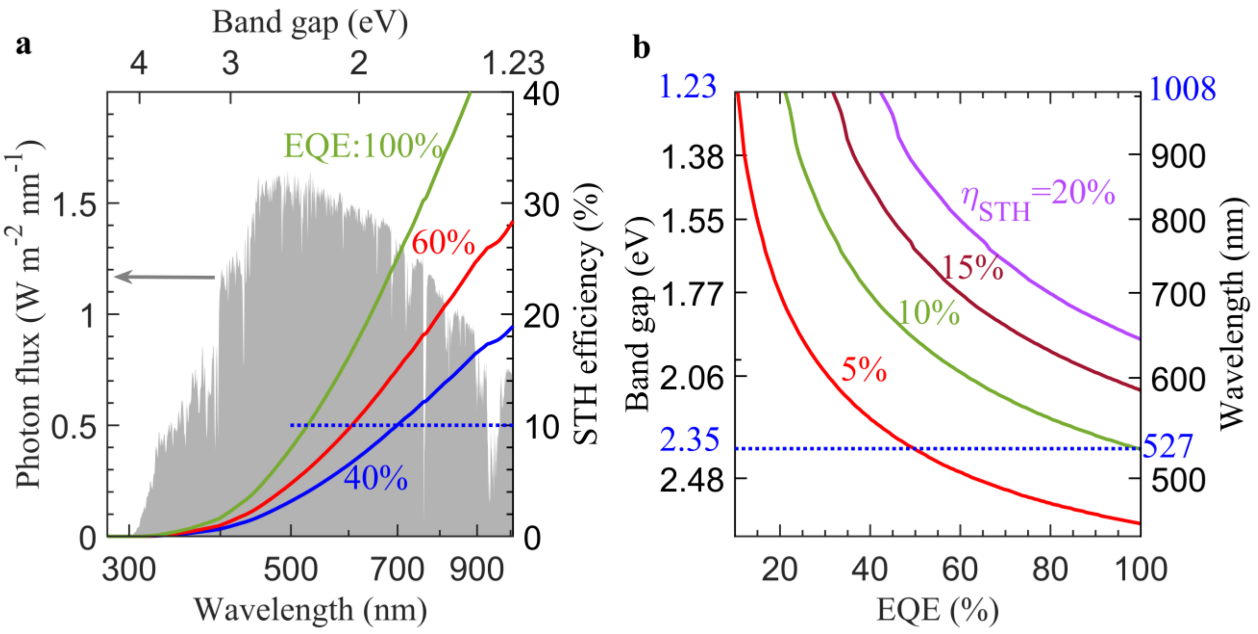

Supplementary Figure 1. Theoretical guidelines to realize highly efficienct photocatalyst for overall water splitting. **a**, Estimated solar-to-hydrogen (STH) efficeincy η_{STH} exhibited from wide range of energy band gap/light absorption edge at external quantum efficiency (EQE).[1] Here, the blue dotted line is the desired $\eta_{\mathrm{STH}} = 10$ % which can be realized from various combination of energy band gap and EQE. **b**, countour plot of the extracted band-gap energy (eV, left axis)/light absorption edge (nm, right axis) and EQE for different STH efficiencies. For STH efficiency >10 %, the band-gap energy of the photocatalytic material should be less than 2.35 eV.

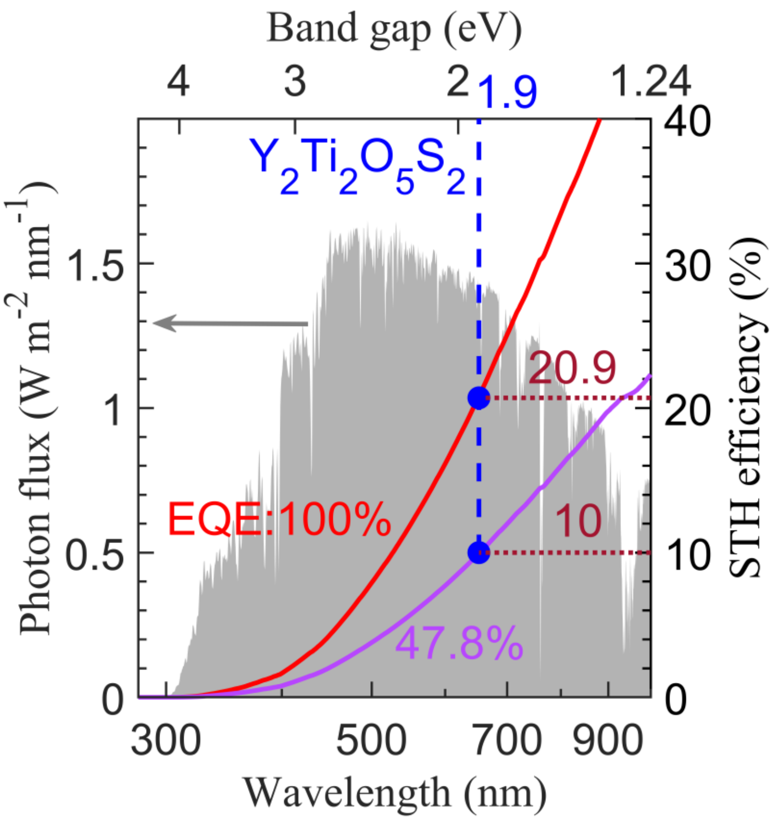

Supplementary Figure 2. Theroretical maximum STH efficiency of $Y_2Ti_2O_5S_2$ photocatalyst for AM 1.5G visible solar spectrum driven overall water splitting.

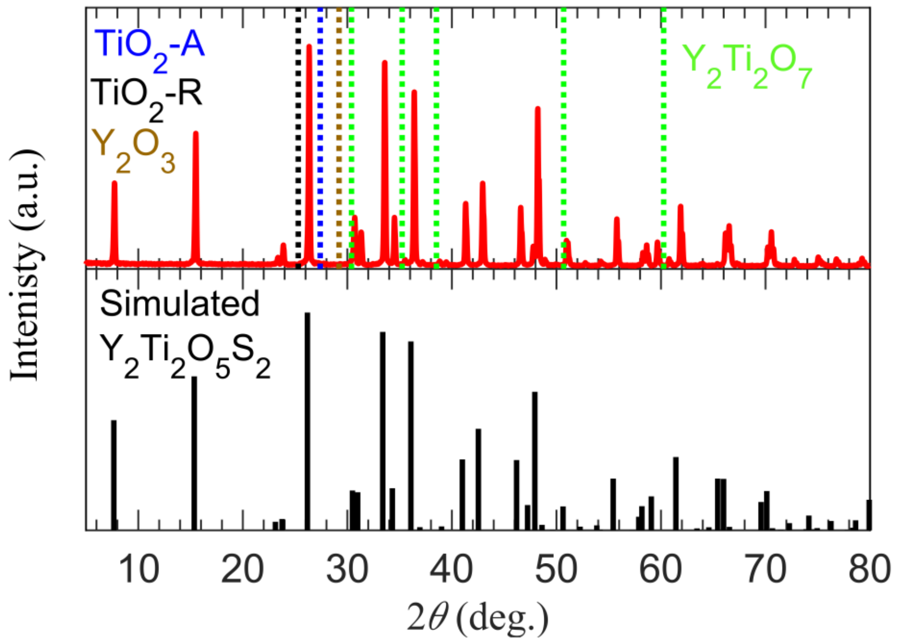

Supplementary Figure 3. XRD pattern (measured- top and simulated- bottom) of solid state synthesised $Y_2Ti_2O_5S_2$ photocatalyst. Dotted lines correspond to the dominant peaks from joint committee

on powder diffraction standard (JCPDS) spectra of TiO_2-anatase (A); JCPDS card no. 21-1272, TiO_2-rutile (R); JCPDS card no. 21-1276, Y_2O_3; JCPDS card no. 88-1040, and $Y_2Ti_2O_7$.[2–4]

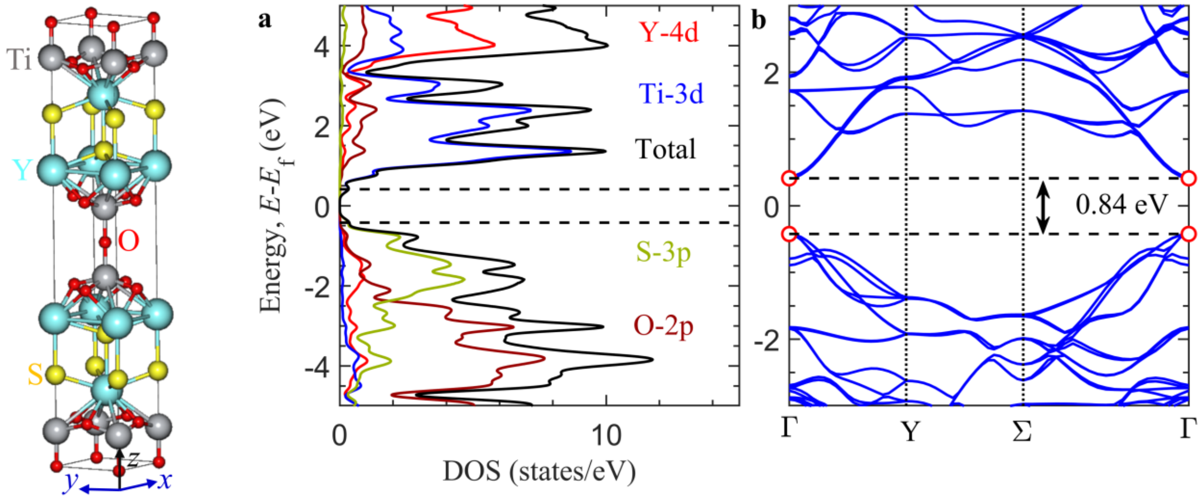

Supplementary Figure 4. Ab-initio first-principle calculations of density functional theory (DFT) of $Y_2Ti_2O_5S_2$ photocatalyst. a, Partial density of states of Y-4d, Ti-3d, S-3p, and O-2p orbitals along with total density of states (in right) of relaxed crystal $Y_2Ti_2O_5S_2$ model. (space group I4/mmm,[5] in left) **b**, Energy band structure indicating underestimated (due to self-interaction errors in exchange correlation function) direct energy band gap of 0.84 eV at Γ point. The dashed lines or open circles at Γ points represent the conduction band (CB) minimum and valence band (VB) maximum of $Y_2Ti_2O_5S_2$.

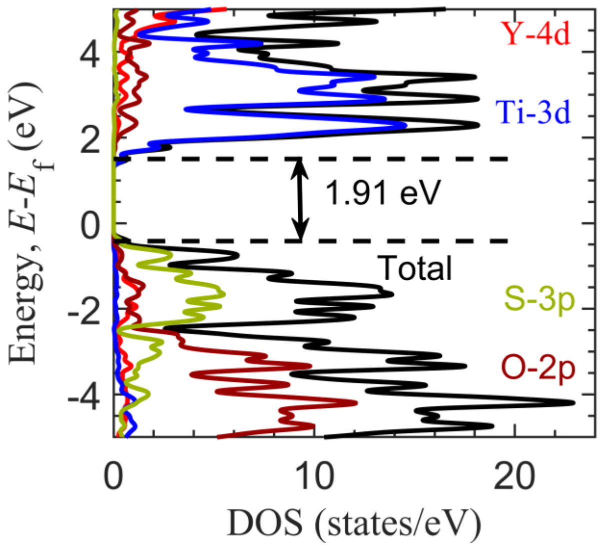

Supplementary Figure 5. Ab-initio first-principle calculations of DFT/HSE06 (HSE06- hybrid functional) for partial density of states of $Y_2Ti_2O_5S_2$ photocatalyst. Computed partial density of states of Y-4d, Ti-3d, S-3p, and O-2p orbitals along with the total density of states of relaxed crystal $Y_2Ti_2O_5S_2$ model (Supplementary Fig. 4a, left). The calculated energy bandgap of 1.91 eV is

consistent with the values measured in Fig. 1c, previously reported experiments,[6] and DFT/HSE06 calculations.[7]

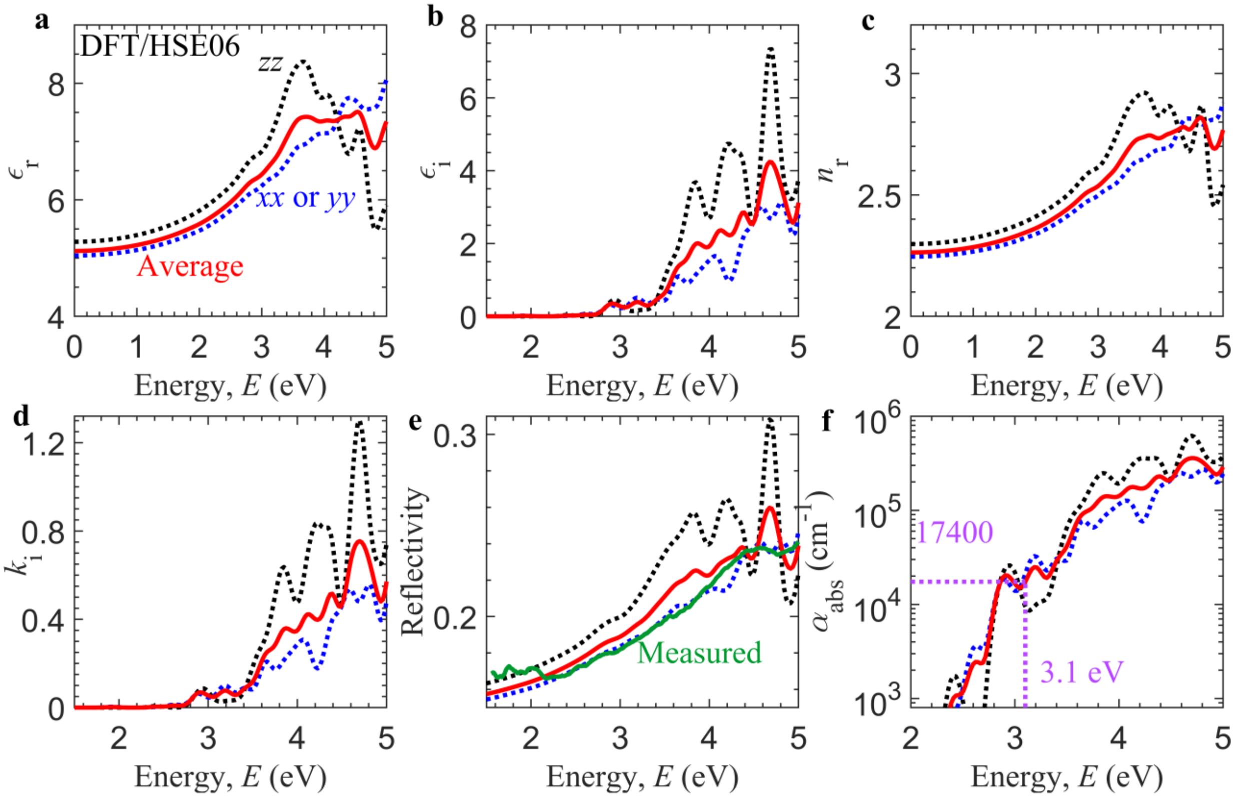

Supplementary Figure 6. Ab-initio first-principle calculations of DFT/HSE06 (HSE06-hybrid functional) for optical properties of $Y_2Ti_2O_5S_2$ photocatalyst. a, b, Simulated real ϵ_r and imaginary ϵ_i components of dielectric function ($\epsilon = \epsilon_r + i\epsilon_i$). **c, d,** Estimated refractive index n_r and extinction coefficient k_i from relations: $2n_r^2 = \left(\epsilon_r^2 + \epsilon_i^2\right)^{1/2} + \epsilon_r$; $2k_i^2 = \left(\epsilon_r^2 + \epsilon_i^2\right)^{1/2} - \epsilon_r$.[8] **e,** Measured and calculated reflectivity. **f,** Absorption coefficient α_{abs} ($= 4\pi E k_i/hc$, where h and c are the Planck constant and the speed of light in vacuum, respectively). Normal reflectivity measurements were performed on the crystal face of particle with the size of 10-20 μm. In panel **e,** the reflectivity is calculated from the simulation results of panels **c** and **d** such that reflectivity $= ((n_r - 1)^2 + k_i^2)/((n_r + 1)^2 + k_i^2)$. The results in panel **e** displays that the measured reflectivity and calculated reflectivity in *xx* or *yy* direction are in excellent agreement, which suggests accurate estimation of absorption coefficient in panel **f**. In panels **a-f,** *xx*, *yy*, and *zz* correspond to the computed optical parameters along *x*, *y*, and *z* directions of $Y_2Ti_2O_5S_2$ crystal model in Supplementary Fig. 4a.

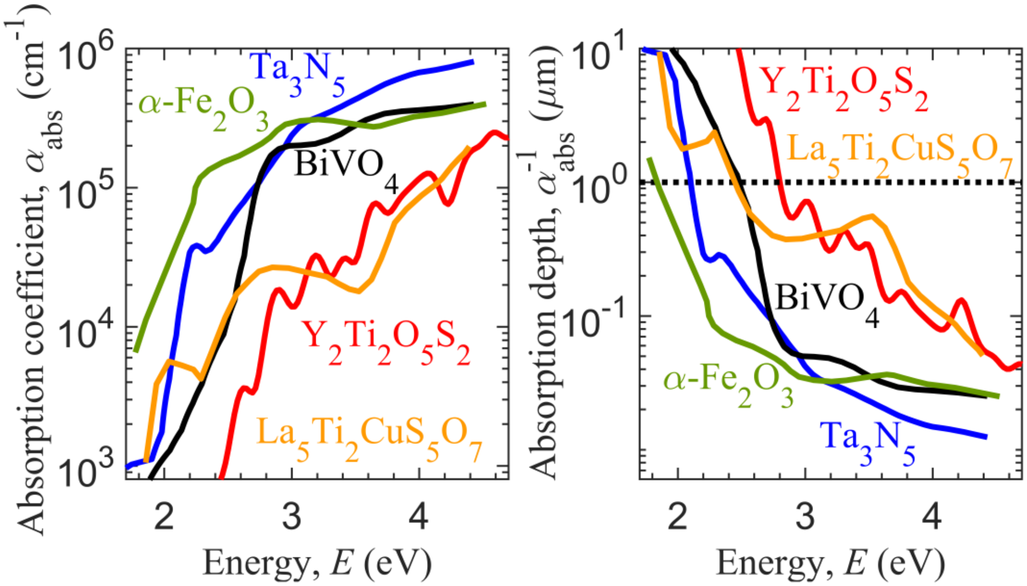

Supplementary Figure 7. Absorption coefficient (left panel) and absorption depth (right panel) of well-studied visible-light absorbing photocatalysts. The absorption depth is estimated from the inverse of absorption coefficient which represents the photocatalyst thickness/depth (from electrolyte interface) required for the absorption of incident solar energy E. In ultraviolet range (E > 3.1 eV), the absorption coefficient α_{abs} of metal oxides ($BiVO_4$, α-Fe_2O_3) and nitride (Ta_3N_5) photocatalysts lies in the range of 10^5- 10^6 cm^{-1}, however, α_{abs} of oxysulfide photocatalysts such as $Y_2Ti_2O_5S_2$ and $La_5Ti_2CuS_5O_7$ ranges almost one order less from 10^4 to 10^5 cm^{-1}. α_{abs} reduces drastically as the energy E decreases towards the energy-band gap (1.9-2.4 eV), present in the visible solar spectrum, of respective photocatalysts. From the right panel, the results display that the $Y_2Ti_2O_5S_2$ and $La_5Ti_2CuS_5O_7$ absorb ultraviolet solar spectrum from 3.1 to 4.2 eV within the depth of 50 to 600 nm, whereas metal oxides and nitride absorb within 10 nm to 50 nm from the electrolyte interface. Owing to low α_{abs}, $Y_2Ti_2O_5S_2$ requires relatively larger particle size to absorb light energy in the range of 1.9 to 3.1 eV than the other prescribed photocatalysts. For instance, the $Y_2Ti_2O_5S_2$ particles with the size of 1 μm can absorb light efficiently up to 2.8 eV, whereas the same size for other photocatalysts has the potentials to absorb up to the band-gap energy. Based on this, it is essential to measure the absorption coefficient to obtain the upper limit of particle size for efficient light absorption. Apart from $Y_2Ti_2O_5S_2$, the α_{abs} spectra for other photocatalysts are obtained from the reported data to highlight the comparison of $Y_2Ti_2O_5S_2$ with other well-explored visible-light-responsive photocatalysts.[9–13]

Transient diffuse reflectance spectroscopy (TDRS)

Supplementary Fig. 8 presents the schematic illustration of transient diffuse reflectance spectroscopy (TDRS) leading to various charge carrier transition processes. Particulate $Y_2Ti_2O_5S_2$ sample is photoexcited by illuminating with pump light of photon energy at 3.1 eV (more than the band-gap energy). This leads to the equal generation of mobile electron density Δn and hole density Δp in CB and VB, respectively. The measured maximum absorption signal $S(0)$ is proportional to the photogenerated charge carrier density (i.e., $S(0) = \beta\Delta n$, here β is proportionality constant) which further depends on the product of pump fluence intensity P_{FL} and absorption coefficient α_{abs},

as per Lambert-Beer law. Charge carriers in respective deep delocalized states of continuum bands (CB and VB) relax towards to the energy levels near band gap. The time scale for such relaxation process is generally within few to hundreds of femtoseconds for semiconducting materials.[14–17] Probe laser pulse of varying energy is employed at different delay time t (with respect to pump excitation pulse) to measure absorption spectrum which is originated from the inter (for high probe photon energy > 1.9 eV) or intra-band (for low probe photon energy < 1.9 eV) transition of photogenerated charge carriers. For instance, in Supplementary Fig. 8, the photogenerated electrons and holes present near band edges absorb probe energy of 0.24 eV and undergo respective intra-band transition.

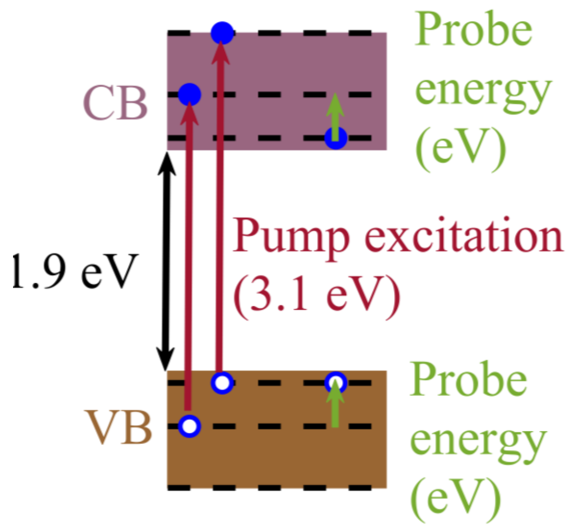

Supplementary Figure 8. Schematic illustration of various inter and intra-band transition of electrons induced by pump-probe method (solid circles- electrons, open circles- holes). The pump photon energy of 3.1 eV excites the $Y_2Ti_2O_5S_2$ photocatalyst (with band-gap energy of 1.9 eV) for inter-band transition to generate electrons and holes in conduction band (CB) and valence band (VB), respectively. The probe photon energy of 0.24 eV leads to intra-band electrons transition in respective bands.

Supplementary Note 1: Estimation of the effect of pump-induced heating on TDR signal

According to previous studies,[9, 18-20] we estimate the temperature change caused by the pump light pulse in this study as follows. If all absorbed energy of pump light is converted to heat, these studies show that the temperature rise can be estimated as

$$\Delta T = Q/\rho C_p,$$

where Q is the absorbed energy of pump light per pulse and volume (J·cm^{-3}), ρ is the molar density (mol·cm^{-3}) of $Y_2Ti_2O_5S_2$, and C_p is the molar heat capacity (J·mol^{-1}·K^{-1}) of $Y_2Ti_2O_5S_2$.

In this study, maximum energy of pump light per pulse is 4.5 μJ and this corresponds to 0.338 mJ·cm^{-2} considering the irradiated area of the pump light. Q is calculated considering the computed absorption coefficient at 3.1 eV (1.74×10^4 cm^{-1}) as

$$Q = 0.338 \times 1.74 \times 10^4 = 5.87 \text{ J·cm}^{-3}$$

ρ is calculated to be 1.02×10^{-2} mol·cm^{-3} from the crystal structure data.[21] C_p of $Y_2Ti_2O_5S_2$ is experimentally unknown. However, if we assume that the Dulong–Petit law for heat capacity of solids can be applied to $Y_2Ti_2O_5S_2$, C_p is estimated to be C_p = 3×R×11= 3×8.31×11=274.4 J·mol^{-1}·K^{-1}, where R is the molar gas constant. Substituting these values into the above equation of ΔT, we obtain ΔT = 2.1 K for maximum energy of pump light per pulse (4.5 μJ/pulse). For the measurement of TDR spectra shown Fig. 1d of the main text, the energy of pump light per pulse is 3.0 μJ/pulse. In this case, ΔT is estimated to be 1.4 K. Thus, the maximum temperature rise is found to be not significant (≤ 2.1 K) in the case of $Y_2Ti_2O_5S_2$.

As highlighted by previous studiess,[9, 18-20] temperature rise causes an energy shift of band gap and broadening of absorption and reflectivity band of the sample, and thermal-induced signal often appears near the band-gap edge and above the band gap and is not observed in energy range far below band-gap energy. In fact, these studies clearly show that transient absorption and reflectivity signals in energy range far below band-gap energies (< 2.2 eV for $BiVO_4$,[9] < 1.7 eV for pentacene,[18] and < 2.0 eV for $LaFeO_3$[20]) are not thermal-induced signals but can be assigned to photoinduced (nonthermal) electronic signals from comparison between temperature-induced differential spectra and the observed pump-induced transient spectra. This means that transient absorption and reflectivity measurements can capture the accurate photoinduced electronic responses when we select proper probe energy range. Therefore, as in these cases, transient signals in energy range far below the band-gap energy (1.9 eV for $Y_2Ti_2O_5S_2$) can be judged to be due to the photoinduced electronic signals in the case of $Y_2Ti_2O_5S_2$.

To further confirm this notion experimentally, we measured steady-state diffuse reflectance spectra of $Y_2Ti_2O_5S_2$ over a temperature range from 296.5 K to 316.5 K using a Fourier transform infrared spectrometer (FT/IR-6100, JASCO) with an optical microscope (IRT-5000, JASCO) and a cryostat (Microstat, Oxford Instruments). Supplementary Fig. 9 shown below is the differential diffuse reflectance spectra defined by $1\text{-}R_T/R_{296.5\text{ K}}$ (%) in the energy range from 0.125 eV to 0. 625 eV. The probe photon energy of 0.24 eV at which we measured detailed kinetics up to 1 μs (Fig. 1f in the main text) is located within this range. As can be seen from the figure, no thermal-induced signal is observed within the range of uncertainty (± 1 %) even when we increase the sample temperature by 20 K from 296.5 K, which is much larger than the above-estimated maximum temperature rise (2.1 K) by pump laser pulse. This result clearly indicates that thermal effect did not reproduce the observed transient spectra shown in Fig. 1e in the main text, and transient spectra from 0.125 eV to 0. 625 eV is surely due to pure photoinduced electronic responses.

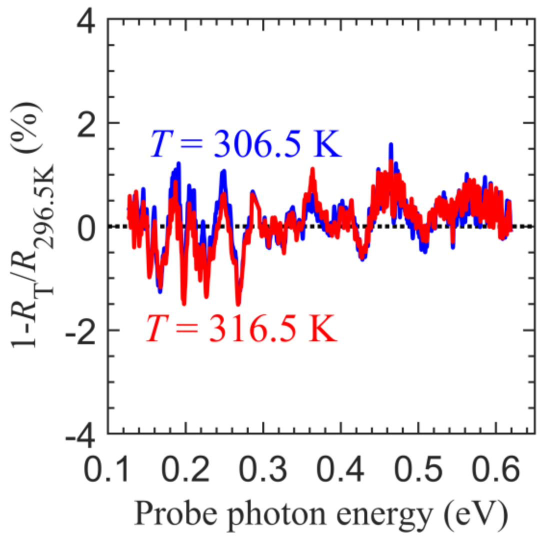

Supplementary Figure 9. Temperature-induced differential diffuse reflectance spectra of $Y_2Ti_2O_5S_2$ in mid-IR range.

Furthermore, as mentioned in the main text, the temporal profiles of absorption signal are found to be almost identical across the entire probe photon energy spectrum (0.15 eV ~ 2.87 eV, Fig. 1e in the main text), indicating that the thermal contribution to the observed transient spectra is quite minor. In addition, we present the TDR kinetics (up to 1 μs) probed at 1.48 eV for various pump fluences in Supplementary Fig. 10. As can be seen, the kinetics feature at 1.48 eV is almost the same as that at 0.24 eV (Fig. 1f in the main text), suggesting that kinetics feature observed at 0.24 eV is universal, independent of probe photon energy.

From above discussion, therefore, it can be concluded that the TDR signal of $Y_2Ti_2O_5S_2$ probed at 0.24 eV reflets only photoinduced electronic processes.

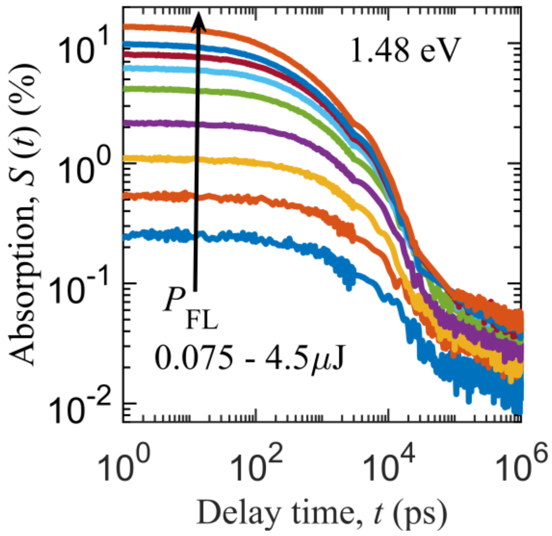

Supplementary Figure 10. TDR kinetics at probe energy of 1.48 eV in $Y_2Ti_2O_5S_2$.

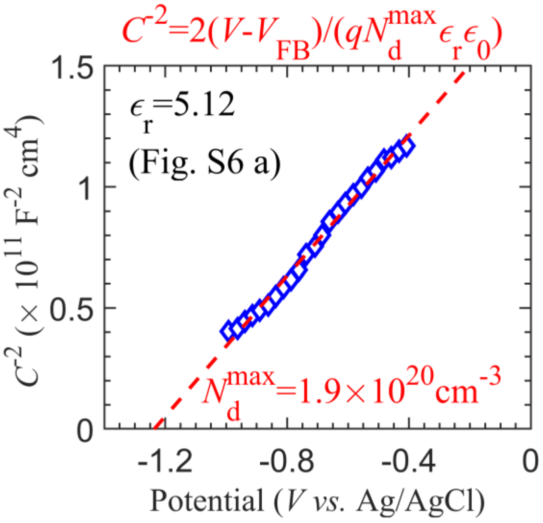

Supplementary Figure 11. Mott-Schottky (MS) analysis of $Y_2Ti_2O_5S_2$ electrode. The reported $Y_2Ti_2O_5S_2$/Ti electrode was prepared by particle-transfer method.[6] Electrochemical impedance (at frequency 1 kHz and AC amplitude 10 mV) was measured in three electrode configurations with Pt and Ag/AgCl as counter and reference electrodes, respectively. An electrolyte solution of 0.1 M Na_2SO_4 was prepared and adjusted by H_2SO_4 (aq.) or NaOH (aq.) for pH of 6.8. The depletion capacitance C at $Y_2Ti_2O_5S_2$ /electrolyte interface reduces with the increase of applied potential V in accordance with the well-known MS relation given by $C^{-2} = 2(V - V_{\mathrm{FB}})/(qN_{\mathrm{d}}^{\mathrm{max}}\epsilon_{\mathrm{r}}\epsilon_0)$. Here, $C, q, \epsilon_0, \epsilon_{\mathrm{r}}, N_{\mathrm{d}}^{\mathrm{max}}$, and V_{FB} are area normalized capacitance, the elementary charge, electrical permittivity of free space, dielectric constant, n-type doping density, and flat band potential, respectively. Using the computed value given by $\epsilon_{\mathrm{r}} = 5.12$ at 0 eV (from Supplementary Fig. 6a), $N_{\mathrm{d}}^{\mathrm{max}} = 1.9 \times 10^{20}$ cm^{-3} is calculated from the slope of linear fit (dashed line) to the measured MS plot. The extracted n-type doping density of $Y_2Ti_2O_5S_2$ is the upper limit as the capacitance was normalized to the projected electrode area which was less than the surface area of $Y_2Ti_2O_5S_2$ /electrolyte interface.

Supplementary Note 2: Model formulation

We consider that $Y_2Ti_2O_5S_2$ photocatalyst is a heavily n-type doped semiconductor (evident from MS analysis, Supplementary Fig. 11), where trapping and detrapping of electrons can be ignored. The energy dispersion of tail states is given by exponential function $g(E) = \exp{(-E/E_0)}/E_0$ such that $\int g(E)dE = 1$, where E is the energy (with respect to VB) in the forbidden energy band gap. Accordingly, the evolution of trapped hole density at energy E with time $p_{\mathrm{t}}(E,t)$ is given by

$$\frac{dp_{\mathrm{t}}(E,t)}{dt} = k_{\mathrm{t}}p_{\mathrm{m}}(t)[N_{\mathrm{t}}g(E) - p_{\mathrm{t}}(E,t)] - k_{\mathrm{d}}p_{\mathrm{t}}(E,t)[N_{\mathrm{m}} - p_{\mathrm{m}}(t)]. \qquad (S1)$$

The first term on the right-hand side of the above equation gives the measure of accumulation of trapped holes which depends on the product of mobile hole density $p_{\mathrm{m}}(t)$ in VB, trapping rate constant k_{t}, and available tail state density for hole capture (difference between tail state density

and trapped hole density at energy E i.e., $N_{\rm t}g(E) - p_{\rm t}(E,t)$). The second term leads to the depletion of trapped holes which is dictated by the product of trapped hole density $p_{\rm t}(E,t)$, detrapping rate constant $k_{\rm d}$, and empty VB states for hole transition (difference between effective density states for VB and mobile hole density i.e., $N_{\rm m} - p_{\rm m}(t)$). Under steady state, $dp_{\rm t}(E,t)/dt = 0$, the trapping and detrapping hole fluxes should be equal and therefore, the detailed balance should hold $k_{\rm d}(E) = k_{\rm t}\exp\,(-E/k_{\rm B}T)$. Besides this, the dynamics of mobile hole density is governed by:

$$\frac{dp_{\rm m}(t)}{dt} = -k_{\rm r}p_{\rm m}(t)n(t) - k_{\rm t}p_{\rm m}(t)[N_{\rm t} - p_{\rm t}(t)] + \int k_{\rm d}(E)p_{\rm t}({\rm E,t})dE[N_{\rm m} - p_{\rm m}(t)]. \qquad (S2)$$

In the above equation, the first term on the right-hand side refers to the bimolecular recombination of mobile hole density $p_{\rm m}(t)$ with electron density $n(t)$ $(= n_{\rm eq} + \Delta p(t)$, considering $\Delta n(t) = \Delta p(t))$. The excess hole density $\Delta p(t)$ is comprised of mobile hole $p_{\rm m}(t)$ and total trapped hole density in VB tail states $p_{\rm t}(t)$, where $p_{\rm t}(t) = \int p_{\rm t}(E,t)dE$. Accordingly, the electron density $n(t) = n_{\rm eq} + p_{\rm m}(t) + p_{\rm t}(t)$. The second term and third term correspond to the total trapping and detrapping of holes across energy E, respectively. Numerical calculations, using Runge-Kutta fourth-order method implemented in MATLAB, are performed to solve Equations (S1) and (S2). Apart from numerical model formulation, for $k_{\rm r} = k_{\rm t}$, we obtain the analytical approximate expression for the time variation of mobile hole $p_{\rm m}^{\rm A}(t)$ and that for trapped hole density $p_{\rm t}^{\rm A}(t)$ in the next section.

Supplementary Note 3: Analytical solutions (Density of mobile $p_{\rm m}^{\rm A}(t)$ and trapped $p_{\rm t}^{\rm A}(t)$ holes)

We study the model shown in Fig. 3d analytically. In this model, the majority carrier concentration is assumed to be high enough so that trap states for the majority carriers are occupied; further trapping of the majority carriers could be ignored. We consider the trapping and detrapping transition processes for minority carriers, and bimolecular band to band recombination of charge carriers. $Y_2Ti_2O_5S_2$ photocatalyst is a heavily n-type doped semiconductor, evident from Supplementary Fig. 11. We consider the trapping and detrapping processes of holes. Exponential tail states of VB, with density N_t and characteristic energy E_0, are introduced. The energy dispersion of tail states is given by exponential function:

$$g(E) = \exp\,(-E/E_0)/E_0 \qquad (S3)$$

such that $\int g(E)dE = 1$, where E is the energy (with respect to VB) in the forbidden energy band gap. The energy dispersion parameter is defined by

$$\alpha = k_{\rm B}T/E_{0,}$$

which decreases by increasing E_0.

Accordingly, the evolution of trapped hole density at energy E with time, $p_{\rm t}(E,t)$, is given by Equation (S1) and the dynamics of mobile hole density, $p_{\rm m}(t)$, is formulated by Equation (S2). Considering $N_{\rm m} > p_{\rm m}(t)$, Equations (S1) and (S2) are simplified as

$$\frac{dp_{\rm t}(E,t)}{dt} = k_{\rm t}p_{\rm m}(t)[N_{\rm t}g(E) - p_{\rm t}(E,t)] - k_{\rm d}(E)N_{\rm m}\,p_{\rm t}(E,t), \qquad (S4)$$

$$\frac{dp_{\mathrm{m}}(t)}{dt} = -k_{\mathrm{r}} p_{\mathrm{m}}(t) n(t) - k_{\mathrm{t}} p_{\mathrm{m}}(t)[N_{\mathrm{t}} - p_{\mathrm{t}}(t)] + \int_0^\infty dE\, k_{\mathrm{d}}(E) N_{\mathrm{m}}\, p_{\mathrm{t}}(E, \mathrm{t}), \qquad (S5)$$

where the detailed balance holds

$$k_{\mathrm{d}}(E) = k_{\mathrm{t}} \exp\left[-\frac{E}{k_{\mathrm{B}} T}\right], \qquad (S6)$$

and the electron density is given by $n(t) = n_{\mathrm{eq}} + \Delta n(t)$ using $\Delta n(t) = \Delta p(t)$ which represents the excess carrier density generated by pulsed light excitation. The excess hole density $\Delta p(t)$ is the sum of the mobile hole $p_{\mathrm{m}}(t)$ and total trapped hole density in VB tail states $p_{\mathrm{t}}(t)$, where $p_{\mathrm{t}}(t) = \int_0^\infty dE\, p_{\mathrm{t}}(E, t)$. Accordingly, the electron density is given by $n(t) = n_{\mathrm{eq}} + p_{\mathrm{m}}(t) + p_{\mathrm{t}}(t)$. For simplicity, we obtain the analytical solutions for $k_r = k_t$. The initial conditions are given by $p_{\mathrm{m}}(0) = \Delta n_0$ and $p_{\mathrm{t}}(E, t) = 0$. We are interested in the late-time asymptotic decay of carrier densities, which are insensitive to the initial conditions; the initial condition for the trapped hole density is assumed to be zero for simplicity.

Equations (S4) and (S5) are nonlinear and will be solved approximately for two stages. In the first stage, we study the kinetics in the absence of detrapping processes. The density of trapped carriers initially increases by trapping of mobile carriers. At long times, the density of trapped carriers decreases by detrapping and recombination processes. As a result, the total density of trapped carriers increases, reaches the maximum and then decreases as shown in Fig. 4a. The processes leading to the maximum in the total density of trapped carriers $p_{\mathrm{t}}(t)$ is termed as the first stage. In the second stage, the total number density of trapped carriers decays from the maximum. The initial distribution of trapped carriers among the trap states at the second stage are obtained from the final distribution of the trapped carriers in the first stage.

Stage 1: By considering initial trapping and recombination processes in the absence of detrapping processes, the kinetic equations for the number density of mobile holes and the number density of occupied trap states can be obtained from Equations (S4) and (S5) as

$$\frac{dp_{\mathrm{t}}^{\mathrm{I}}(E, t)}{dt} = k_{\mathrm{t}} p_{\mathrm{m}}^{\mathrm{I}}(t)\left[N_{\mathrm{t}} g(E) - p_{\mathrm{t}}^{\mathrm{I}}(E, t)\right], \qquad (S7)$$

and

$$\frac{dp_{\mathrm{m}}^{\mathrm{I}}(t)}{dt} = -k_{\mathrm{t}} p_{\mathrm{m}}^{\mathrm{I}}(t)[n_{\mathrm{eq}} + p_{\mathrm{m}}^{\mathrm{I}}(t)] - k_{\mathrm{t}} p_{\mathrm{m}}^{\mathrm{I}}(t) N_{\mathrm{t}}, \qquad (S8)$$

where we approximated $n(t) = n_{\mathrm{eq}} + p_{\mathrm{m}}^{\mathrm{I}}(t) + p_{\mathrm{t}}^{\mathrm{I}}(t) \approx n_{\mathrm{eq}} + p_{\mathrm{m}}^{\mathrm{I}}(t)$ and $N_{\mathrm{t}} - p_{\mathrm{t}}^{\mathrm{I}}(t) \approx N_{\mathrm{t}}$ .The solution of Equation (S8) with the initial condition given by $p_m^{\mathrm{I}}(0) = \Delta n_0$ is obtained as

$$p_{\mathrm{m}}^{\mathrm{I}}(t) = \frac{\Delta n_0 \left(N_{\mathrm{t}} + n_{\mathrm{eq}}\right)}{\exp\left(k_{\mathrm{t}}\left(N_{\mathrm{t}} + n_{\mathrm{eq}}\right)t\right)\left(N_{\mathrm{t}} + n_{\mathrm{eq}} + \Delta n_0\right) - \Delta n_0}$$

$$= \sum_{j=0}^{\infty} \exp[-k_{\mathrm{t}}\left(N_{\mathrm{t}} + n_{\mathrm{eq}}\right)(j+1)t] \frac{\left(N_{\mathrm{t}} + n_{\mathrm{eq}}\right)\Delta n_0{}^{j+1}}{\left(N_{\mathrm{t}} + n_{\mathrm{eq}} + \Delta n_0\right)^{j+1}}\ , \qquad (S9)$$

where we multiply $\exp\left(-k_{\mathrm{t}}\left(N_{\mathrm{t}}+n_{\mathrm{eq}}\right)t\right)$ to the numerator and the denominator of the first line of Equation (S9) and use $1/(a-b)=\sum_{j=0}^{\infty}(b/a)^j$. By time integration of Equation (S9), we obtain

$$k_{\mathrm{t}}\int_0^{\infty} dt\, p_{\mathrm{m}}^{\mathrm{I}}(t)=\sum_{j=0}^{\infty}\frac{{\Delta n_0}^{j+1}}{(j+1)\left(N_{\mathrm{t}}+n_{\mathrm{eq}}+\Delta n_0\right)^{j+1}}$$

$$=-\log\left(\frac{N_{\mathrm{t}}+n_{\mathrm{eq}}}{N_{\mathrm{t}}+n_{\mathrm{eq}}+\Delta n_0}\right), \qquad (S10)$$

where we used $\sum_{j=0}^{\infty}x^{j+1}/(j+1)=\int_0^x dy\sum_{j=0}^{\infty}y^j=\int_0^x dy\,1/(1-y)=-\log|1\text{-x}|$. The solution of Equation (S7) is obtained from $dp_{\mathrm{t}}^{\mathrm{I}}(E,t)/\left(N_{\mathrm{t}}g(E)-p_{\mathrm{t}}^{\mathrm{I}}(E,t)\right)=dtk_{\mathrm{t}}p_{\mathrm{m}}^{\mathrm{I}}(t)$ as

$$p_{\mathrm{t}}^{\mathrm{I}}(E,t)=N_{\mathrm{t}}g(E)\left[1-\exp\left(-\int_0^t dt_1\, k_{\mathrm{t}}p_{\mathrm{m}}^{\mathrm{I}}(t_1)\right)\right] \qquad (\mathrm{S11})$$

By taking the limit of $t\to\infty$ and substituting Equation (S10), Equation (S11) becomes

$$p_{\mathrm{t}}^{\mathrm{I}}(E,\infty)=p(0)g(E) \qquad (S12)$$

where $p(0)$ is given by

$$p(0)=\frac{\Delta n_0 N_{\mathrm{t}}}{N_{\mathrm{t}}+n_{\mathrm{eq}}+\Delta n_0}. \qquad (S13)$$

Equations (S12) and (S13) will be used as the initial condition for stage 2.

Stage 2: Initially, the number density of trapped carriers increases. After the initial time range where the initial trapping dominates, the number density of trapped carriers reaches the maximum and turns to decrease by the recombination of detrapped holes. We analyse the decay process using the initial conditions determined from Equation (S12) as

$$p_{\mathrm{t}}(E,0)=p(0)g(E), \qquad (S14)$$

where $p(0)$ is given by Equation (S13).

At long times, $p_{\mathrm{m}}(t)$ decays but $N_{\mathrm{t}}+n_{\mathrm{eq}}$ is time-independent; Equation (S5) can be linearized by ignoring $p_{\mathrm{m}}(t)$ with respect to $N_{\mathrm{t}}+n_{\mathrm{eq}}$,

$$\frac{dp_{\mathrm{m}}(t)}{dt}=-k_{\mathrm{t}}p_{\mathrm{m}}(t)\left(N_{\mathrm{t}}+n_{\mathrm{eq}}\right)+\int k_{\mathrm{d}}(E)N_{\mathrm{m}}p_{\mathrm{t}}(E,\mathrm{t})dE. \qquad (S15)$$

The solution can be expressed as

$$p_{\mathrm{m}}(t)=G(t)\Delta n_0+\int_0^t dt_1\, G(t-t_1)\int_0^{\infty} dE\, k_{\mathrm{d}}(E)N_{\mathrm{m}}p_{\mathrm{t}}(E,t_1), \qquad (S16).$$

where we defined the time-evolution kernel by $G(t)=\exp\left[-k_{\mathrm{t}}\left(N_{\mathrm{t}}+n_{\mathrm{eq}}\right)t\right]$. At long times, the contribution from the first term on the right-hand side can be small compared to the second term. By assuming time scale separation of trapping and that of detrapping, $p_{\mathrm{t}}(E,t_1)$ can be replaced by

$p_{\mathrm{t}}(E,t)$; the decay of $G(t)$ is too fast compared to that of $p_{\mathrm{t}}(E,t)$ during $t-t_1$. The second term can be further simplified by decoupling time convolution as

$$p_{\mathrm{m}}(t)=\frac{1-\exp\left[-k_{\mathrm{t}}\left(N_{\mathrm{t}}+n_{\mathrm{eq}}\right)t\right]}{k_t\left(N_t+n_{eq}\right)}\int_0^\infty dE\, k_{\mathrm{d}}(E)N_{\mathrm{m}}p_{\mathrm{t}}(E,t)$$

$$\approx\frac{1}{k_{\mathrm{t}}\left(N_{\mathrm{t}}+n_{\mathrm{eq}}\right)}\int_0^\infty dE\, k_{\mathrm{d}}(E)N_{\mathrm{m}}p_{\mathrm{t}}(E,t). \quad (S17)$$

By substituting Equation (S17) into Equation (S4), we obtain

$$\frac{dp_{\mathrm{t}}(E,t)}{dt}=\frac{N_{\mathrm{t}}g(E)-p_{\mathrm{t}}(E,t)}{N_{\mathrm{t}}+n_{\mathrm{eq}}}\int_0^\infty dE\, k_{\mathrm{d}}(E)N_{\mathrm{m}}p_{\mathrm{t}}(E,\mathrm{t})-k_{\mathrm{d}}(E)N_{\mathrm{m}}\, p_{\mathrm{t}}(E,t). \quad (S18)$$

Equation (S18) can be regarded as the generalization of multiple trapping model of intrinsic semiconductors to n-type doped semiconductors.[22] We integrate Equation (S18) over E and find,

$$\frac{dp_{\mathrm{t}}(t)}{dt}=-\frac{p_{\mathrm{t}}(t)+n_{\mathrm{eq}}}{N_{\mathrm{t}}+n_{\mathrm{eq}}}\int_0^\infty dE\, k_{\mathrm{d}}(E)N_{\mathrm{m}}p_{\mathrm{t}}(\mathrm{E},\mathrm{t}), \quad (S19)$$

where, $p_{\mathrm{t}}(t)=\int_0^\infty dE\, p_{\mathrm{t}}(E,t)$. We introduce the normalized distribution function of trapped carriers given by

$$\phi(E,t)=p_{\mathrm{t}}(E,t)/\left(p_{\mathrm{t}}(t)+n_{\mathrm{eq}}\right). \quad (S20)$$

Equation (S19) can be rewritten as

$$\frac{dp_{\mathrm{t}}(t)}{dt}=-\frac{\left(p_{\mathrm{t}}(t)+n_{\mathrm{eq}}\right)^2}{N_{\mathrm{t}}+n_{\mathrm{eq}}}\Phi(t), \quad (S21)$$

where we defined

$$\Phi(t)=\int_0^\infty dE\, k_{\mathrm{d}}(E)N_{\mathrm{m}}\phi(E,t). \quad (S22)$$

Similarly, Equation (S18) can be rewritten as

$$\frac{d\phi(E,t)}{dt}=-k_{\mathrm{d}}(E)N_{\mathrm{m}}\phi(E,t)+\frac{N_{\mathrm{t}}g(E)}{N_{\mathrm{t}}+n_{\mathrm{eq}}}\Phi(t). \quad (S23)$$

The initial condition for $\phi(E,t)$ can be obtained using Equation (S14) as

$$\phi(E,0)=\phi_0\, g(E), \quad (S24)$$

where ϕ_0 is expressed as

$$\phi_0=p_{\mathrm{t}}(0)/\left(p_{\mathrm{t}}(0)+n_{\mathrm{eq}}\right)=\frac{1}{1+n_{\mathrm{eq}}\left(N_{\mathrm{t}}+n_{\mathrm{eq}}+\Delta n_0\right)/(\Delta n_0 N_{\mathrm{t}})}. \quad (S25)$$

By integrating Equation (S21), we obtain

$$\frac{1}{p_{\mathrm{t}}(t)+n_{\mathrm{eq}}}=\frac{1}{p_{\mathrm{t}}(0)+n_{\mathrm{eq}}}+\frac{1}{N_{\mathrm{t}}+n_{\mathrm{eq}}}R(t), \tag{S26}$$

where $R(t)$ is defined by

$$R(t)=\int_0^t dt_1\,\Phi(t_1). \tag{S27}$$

The Laplace transform of $R(t)$can be expressed as

$$\widehat{R}(s)=\widehat{\Phi}(s)/s. \tag{S28}$$

Now, we calculate $\widehat{\Phi}(s)$. By applying Laplace transform on Equation (S23), we obtain

$$\widehat{\phi}(E,s)=\frac{g(E)}{s+k_{\mathrm{d}}(E)N_{\mathrm{m}}}\left(\phi_0+\frac{\widehat{\Phi}(s)}{1+n_{\mathrm{eq}}/N_{\mathrm{t}}}\right). \tag{S29}$$

We multiply $k_{\mathrm{d}}(E)N_{\mathrm{m}}$ on both sides of Equation (S29) and integrate over E. After rearrangement, $\widehat{\Phi}(s)$ can be expressed as[22]

$$\widehat{\Phi}(s)=\phi_0\left(1+\frac{n_{\mathrm{eq}}}{N_{\mathrm{t}}}\right)\left(\frac{1+n_{\mathrm{eq}}/N_{\mathrm{t}}}{\widehat{h}(s)+n_{\mathrm{eq}}/N_{\mathrm{t}}}-1\right) \tag{S30}$$

$$\approx\phi_0\frac{N_{\mathrm{t}}+n_{\mathrm{eq}}}{n_{\mathrm{eq}}}\left(1-\frac{N_{\mathrm{t}}+n_{\mathrm{eq}}}{n_{\mathrm{eq}}}\widehat{h}(s)\right), \tag{S31}$$

where $\widehat{h}(s)$ is given using $\int_0^\infty dE\,g(E)=1$ as

$$\widehat{h}(s)=1-\int_0^\infty dE\frac{k_{\mathrm{d}}(E)N_{\mathrm{m}}g(E)}{s+k_{\mathrm{d}}(E)N_{\mathrm{m}}}=\int_0^\infty dE\frac{sg(E)}{s+k_{\mathrm{d}}(E)N_{\mathrm{m}}}, \tag{S32}$$

and Equation (S31) is the asymptotic expression derived using $\widehat{h}(s)\rightarrow 0$ as $s\rightarrow 0$. By substituting Equations (S3) and (S6), Equation (S32) can be expressed as

$$\widehat{h}(s)={}_2F_1(1,\alpha;1+\alpha,-k_{\mathrm{t}}N_{\mathrm{m}}/s)\approx\frac{\pi\alpha}{\sin(\pi\alpha)}\left(\frac{s}{k_{\mathrm{t}}N_{\mathrm{m}}}\right)^{\alpha}, \tag{S33}$$

where, ${}_2F_1(1,\alpha;1+\alpha,-k_{\mathrm{t}}N_{\mathrm{m}}/s)$ indicates the Gauss hypergeometric function and the leading order terms of $\widehat{h}(s)$ in the limit of small s is shown.[22,23] By substituting Equation (S33) into Equation (S31), we obtain $\widehat{\Phi}(s)$ in Equation (S2). By the inverse-Laplace transform of Equation (S28), we obtain

$$\mathrm{R}(t)=\phi_0\frac{N_{\mathrm{t}}+n_{\mathrm{eq}}}{n_{\mathrm{eq}}}\left(1-\frac{N_{\mathrm{t}}+n_{\mathrm{eq}}}{n_{\mathrm{eq}}}\frac{\sin(\pi\alpha)}{\Gamma(1-\alpha)\pi\alpha(k_tN_mt)^{\alpha}}\right), \tag{S34}$$

where $\Gamma(x)$ denotes the gamma function.[23] By substituting Equation (S34) into Equation (S26), the asymptotic decay is obtained as

$$p_t(t)\approx A_{\mathrm{asym}}/t^{\alpha}, \tag{S35}$$

where A_{asym} is given by

$$A_{asym} = \frac{N_t \pi \alpha}{[1 + (n_{eq}/\Delta n_0)]\Gamma(1-\alpha)\, sin(\pi\alpha)\, (k_t N_m)^{\alpha}} \quad (S36)$$

and ϕ_0 given by Equation (S25) is substituted. Equation (S35) is equal to Equation (2) in the main manuscript. We determine the exponent α by fitting the experimental data on asymptotic decay to the power law given by Equation (S35). We, then, analyze the amplitude of the power law obtained from the power-law fitting using Equation (S36). By using the α value thus determined together with $k_t N_m$ and n_{eq} determined by analyzing the initial decay kinetics up to 3 ns (stage 1), we find N_t by studying the amplitude of the power-law decay as a function of Δn_0; Δn_0can be varied by changing the excitation light intensity.

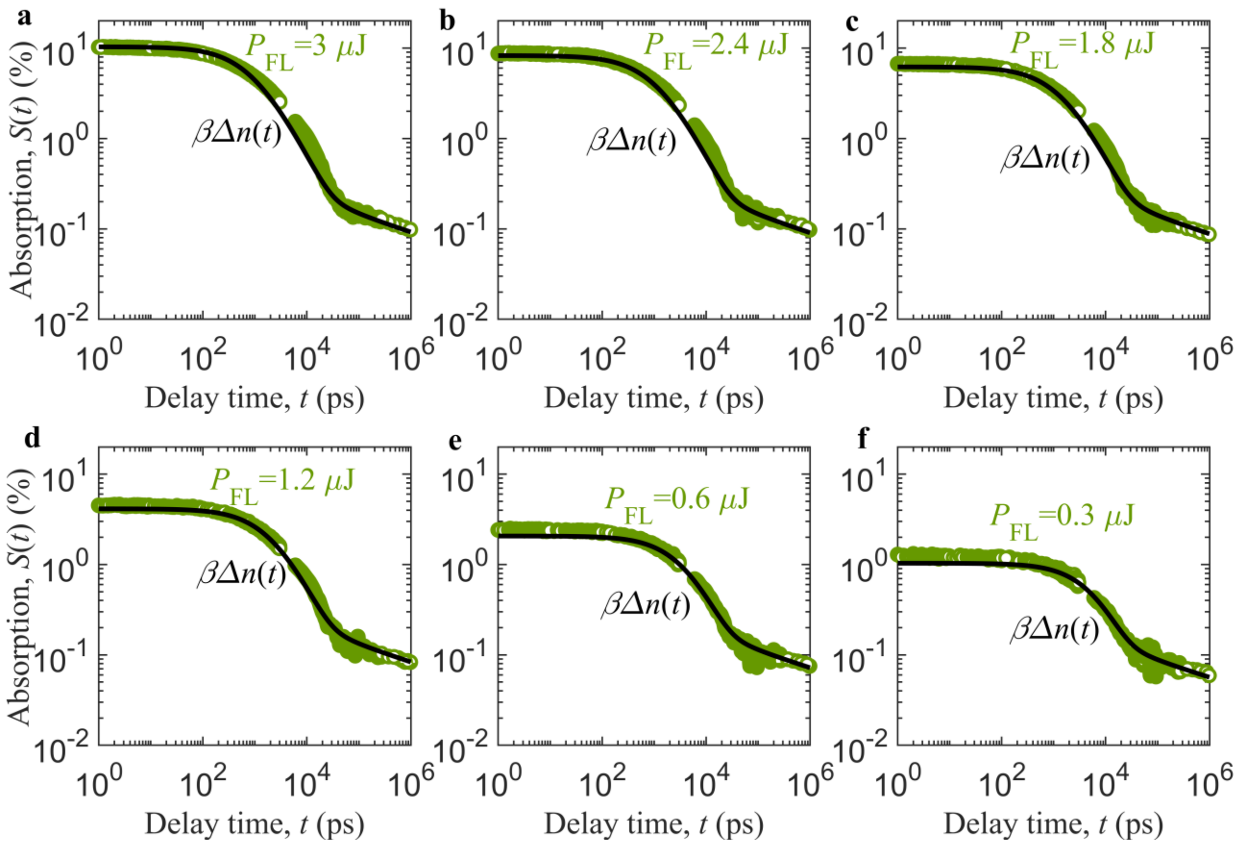

Supplementary Figure 12. Transient diffuse reflectance spectroscopy of $Y_2Ti_2O_5S_2$ photocatalyst. **a-f**, Pump fluence dependence of measured (circle symbol) and simulated (solid line) absorption decay with probe delay time *t*. The excitation pump fluence intensity P_{FL} with photon energy of 3.1 eV is varied and the probe photon energy is fixed to 0.24 eV. The simulated electron density is scaled to absorption using $S(t) = \beta\Delta n(t)$, where $\beta = 1.30 \times 10^{-18}$ cm^3 which is extracted from Fig. 2b of the main text.

Supplementary Table 1. Parameters employed for the developed model of $Y_2Ti_2O_5S_2$ photocatalyst.

Parameters, symbols	Numerical value
Effective density of states for CB/VB, N_{m}	$10^{20}\mathrm{cm}^{-3}$ [a]
Absorption coefficient, α_{abs}	$17400\ \mathrm{cm}^{-1}$ [b]
Excess electron and hole density for Pump fluence intensity $P_{\mathrm{FL}} = 3\ \mu\mathrm{J}$, $\Delta n_0 = n(0) = p_{\mathrm{m}}(0)$	$7.90 \times 10^{18}\ \mathrm{cm}^{-3}$ [c]
Pump fluence intensity, P_{FL}	$0.075 - 4.5\ \mu\mathrm{J}$ [d]
Proportionality constant, β	$1.30 \times 10^{-18}\ \mathrm{cm}^3$ [e]
Bimolecular/trapping rate constant, $k_{\mathrm{r}} = k_{\mathrm{t}}$	$1.57 \times 10^{-10}\ \mathrm{cm}^3\mathrm{s}^{-1}$ [f]
n-type doping density, n_{eq}	$5.2 \times 10^{17}\ \mathrm{cm}^{-3}$ [f, g]
Trap density of VB tail states, N_{t}	$5.1 \times 10^{17}\ \mathrm{cm}^{-3}$ [f, g]
Dispersion parameter, α	0.19 [g]
Characteristic energy of VB tail states, $E_0 = \frac{k_{\mathrm{B}}T}{\alpha}$	0.137 eV [g]

[a] Typical values of direct energy band gap semiconducting materials.[24]

[b] Computed from DFT/HSE06 calculations in Supplementary Fig. 6.

[c] Estimated from Lambert-Beer law for given pump fluence intensity P_{FL}.

[d] Experimental tuning of pump fluence intensity to obtain corresponding Δn_0.

Estimated from [e] Fig. 2(b), [f] early-time decay (Fig. 2), and [g] late-time decay analysis (Fig. 3) of transient diffuse reflectance spectroscopy in the main text.

Supplementary Note 4: Impact of particle size on internal quantum efficiency

By assuming uniform photoexcited carrier generation inside spherical particles of $Y_2Ti_2O_5S_2$ with uniform coverage of reactive sites on the surface of particles, the internal quantum efficiency (IQE) can be estimated by solving the diffusion equation under the influence of recombination and trapping in the steady state,

$$0 = D\nabla^2 p_{\mathrm{m}}(r) - k_{\mathrm{eff}} p_{\mathrm{m}}(r) + G. \qquad (S37)$$

Here, r is the distance from the origin or center of the spherical particle, D is the diffusion constant of mobile carriers, $k_{\mathrm{eff}} = k_{\mathrm{r}} n_{\mathrm{eq}} + k_{\mathrm{t}} N_{\mathrm{t}}$ is the effective carrier decay rate owing to recombination, G is the carrier generation rate per unit volume and trapping and $n_{\mathrm{eq}} \gg \Delta n_0$ under AM 1.5G operating condition is assumed. The particle radius is denoted by R and the density of mobile carriers at R is set zero by assuming infinitely fast limit of the surface charge extraction rate constant. The probability of finding the mobile carriers at r is given by[25]

$$p_{\mathrm{m}}(r) = \frac{G}{k_{\mathrm{eff}}}\left(1 - \frac{R}{r}\right)\frac{\mathrm{Sinh}\left(\frac{r}{L_{\mathrm{D}}}\right)}{\mathrm{Sinh}(R/L_{\mathrm{D}})}, \qquad (S38)$$

where the diffusion length is defined as $L_{\mathrm{D}} = \sqrt{D/k_{\mathrm{eff}}}$. The diffusion limited charge extraction rate at the surface is obtained as

$$R_{\mathrm{ex}} = -4\pi D R^2 \frac{\partial}{\partial r} p_{\mathrm{m}}(r) = 4\pi R^2 L_{\mathrm{D}} \left[\coth\left(\frac{R}{L_{\mathrm{D}}}\right) - \frac{L_{\mathrm{D}}}{R}\right] G \quad \text{at} \qquad r = R \ . \qquad (S39)$$

The IQE for the uniform photo-excited carrier generation inside spherical particles (Equation (3) of main text) is obtained as

$$IQE = R_{\mathrm{ex}} / \left[\left(\frac{4\pi}{3}\right) R^3 G\right] = \frac{3L_{\mathrm{D}}}{R}\left[\coth\left(\frac{R}{L_{\mathrm{D}}}\right) - \frac{L_{\mathrm{D}}}{R}\right] \ . \qquad (S40)$$

Equation (S40) is derived by ignoring recombination between holes and excess electron density over n_{eq} and assuming ideally fast carrier extraction rate at the surface for gas evolution reactions. Nevertheless, the ideal IQE values against the particle size ($L_{\mathrm{avg}} = 2R$) can be estimated from Equation (S40).

Supplementary Note 5: Quantum efficiency

We consider the IQE in the steady states, where the optical excitation at a rate G is applied and the particle size is optimized. Here, we take into account the recombination between holes and excess electron density over n_{eq} and remove the assumption of ideally fast carrier extraction rate at the surface for gas evolution reactions. Since the gas production rate is proportional to the mobile minority carrier density p_{m} with the carrier extraction rate k_{et} , from Equations (S1) and (S2), we obtain

$$0 = k_{\mathrm{t}} p_{\mathrm{m}} [N_{\mathrm{t}} g(E) - p_{\mathrm{t}}(E)] - k_{\mathrm{d}}(E) N_{\mathrm{m}}\, p_{\mathrm{t}}(E), \qquad (S41)$$

$$0 = G - k_{\mathrm{et}} p_{\mathrm{m}} - k_{\mathrm{r}} p_{\mathrm{m}} n - k_{\mathrm{t}} p_{\mathrm{m}} [N_{\mathrm{t}} - p_{\mathrm{t}}] + \int_0^\infty dE\, k_{\mathrm{d}}(E) N_{\mathrm{m}}\, p_{\mathrm{t}}(\mathrm{E}), \qquad (S42)$$

where the time-dependence is removed from notations under steady state condition and $n = n_{\mathrm{eq}} + p_{\mathrm{m}} + p_{\mathrm{t}}$ and $p_{\mathrm{t}} = \int_0^\infty dE\, p_{\mathrm{t}}(E)$. From Equation (S41), we express the trapped hole density at different energy E as

$$p_{\mathrm{t}}(E) = \frac{k_{\mathrm{t}} p_{\mathrm{m}}}{k_{\mathrm{t}} p_{\mathrm{m}} + k_{\mathrm{d}}(E) N_{\mathrm{m}}} N_{\mathrm{t}} g(E) \ . \qquad (S43)$$

By substituting Equation (S43) into Equation (S41) and integrating over E, we obtain an equation for $\int_0^\infty dE\, k_{\mathrm{d}}(E) N_{\mathrm{m}}\, p_{\mathrm{t}}(\mathrm{E})$ as

$$N_{\mathrm{t}} - p_{\mathrm{t}} = N_{\mathrm{t}} \int_0^\infty dE \frac{k_{\mathrm{d}}(E) N_{\mathrm{m}} g(E)}{k_{\mathrm{t}} p_{\mathrm{m}} + k_{\mathrm{d}}(E) N_{\mathrm{m}}} \ , \qquad (S44)$$

where we used $p_{\mathrm{t}} = \int_0^\infty dE\, p_{\mathrm{t}}(E)$.The right-hand side can be evaluated by using $k_{\mathrm{d}}(E) N_{\mathrm{m}} / (k_{\mathrm{t}} p_{\mathrm{m}} + k_{\mathrm{d}}(E) N_{\mathrm{m}}) = 1 - k_{\mathrm{t}} p_{\mathrm{m}} / (k_{\mathrm{t}} p_{\mathrm{m}} + k_{\mathrm{d}}(E) N_{\mathrm{m}})$ and $\int_0^\infty dE g(E) = 1$; Equation (S44) is simplified as

$$p_{\mathrm{t}} = N_{\mathrm{t}} \hat{h}(k_{\mathrm{t}} p_{\mathrm{m}}) \approx N_{\mathrm{t}} \frac{\pi\alpha}{\sin(\pi\alpha)} \left(\frac{p_{\mathrm{m}}}{N_{\mathrm{m}}}\right)^{\alpha}, \qquad (S45)$$

where $\hat{h}(s)$ is defined by Equation (S32) and simplified by using Equation (S33). By combining Equations (S42) and (S44), we find $G = k_{\mathrm{et}} p_{\mathrm{m}} + k_{\mathrm{r}} p_{\mathrm{m}} n$, which indicates that the photogenerated carriers should either be extracted to produce gas or recombine. By substituting Equation (S45) in $G = k_{\mathrm{et}} p_{\mathrm{m}} + k_{\mathrm{r}} p_{\mathrm{m}} n$, we obtain

$$G = p_{\mathrm{m}}\left(k_{\mathrm{et}} + k_{\mathrm{r}}n_{\mathrm{eq}} + k_{\mathrm{r}}N_{\mathrm{t}}\frac{\pi\alpha}{\sin(\pi\alpha)}\left(\frac{p_{\mathrm{m}}}{N_{\mathrm{m}}}\right)^{\alpha} + k_{\mathrm{r}}p_{\mathrm{m}}\right). \tag{S46}$$

The internal quantum efficiency can be obtained from $IQE = k_{\mathrm{et}}p_{\mathrm{m}}/G$. In the limit of weak excitation light intensity, we find,

$$IQE = k_{\mathrm{et}}/\left(k_{\mathrm{et}} + k_{\mathrm{r}}n_{\mathrm{eq}}\right). \tag{S47}$$

At the intermediate light intensity satisfying the condition given by

$$N_{\mathrm{m}}\left(\frac{\sin(\pi\alpha)}{\pi\alpha}\frac{k_{\mathrm{et}} + k_{\mathrm{r}}n_{\mathrm{eq}}}{k_{\mathrm{r}}N_{\mathrm{t}}}\right)^{1/\alpha} < p_{\mathrm{m}}, \tag{S48}$$

and

$$p_{\mathrm{m}} < \left(\frac{\pi\alpha}{\sin(\pi\alpha)}\frac{N_{\mathrm{t}}}{N_{\mathrm{m}}^{\alpha}}\right)^{1/(1-\alpha)}, \tag{S49}$$

we obtain

$$IQE = k_{\mathrm{et}}\left(\frac{\sin(\pi\alpha)}{\pi\alpha}\frac{N_{\mathrm{m}}^{\alpha}}{k_{\mathrm{r}}N_{\mathrm{t}}G^{\alpha}}\right)^{1/(1+\alpha)}. \tag{S50}$$

By substituting the values from Supplementary Table 1 into Equation (S48), we find $0.83 < p_{\mathrm{m}}/N_{\mathrm{m}}$, which is unlikely satisfied due to the high value of n_{eq}. If the value of n_{eq} is decreased 100-fold, we find $2.5 \times 10^{-11} < p_{\mathrm{m}}/N_{\mathrm{m}}$from Equation (S48) and $p_{\mathrm{m}}/N_{\mathrm{m}} < 0.0016$ from Equation (S49). Therefore, if n_{eq} is decreased 100-fold, the internal quantum efficiency could show the light intensity dependence when the light intensity is varied below the air mass (AM) 1.5G sunlight illumination intensity. In the limit of strong light intensity satisfying $(p_{\mathrm{m}}/N_{\mathrm{m}})^{\alpha} < p_{\mathrm{m}}/N_{\mathrm{t}}$, we obtain $G \approx k_{\mathrm{r}}{p_{\mathrm{m}}}^2$ and $IQE = k_{\mathrm{et}}/(k_{\mathrm{r}}p_{\mathrm{m}})$.

In Fig. 5b and Supplementary Fig. 11, We show IQE as a function of G obtained from Equation (S46), where the values in Supplementary Table 1 are used, together with the cases when the values of n_{eq} are reduced. We set (a) $k_{\mathrm{et}} = 1.0 \times 10^{7}\ \mathrm{s}^{-1}$ and (b) $1.0 \times 10^{6}\ \mathrm{s}^{-1}$ to satisfy $IQE = 10.14\ \%$ and $IQE = 1.1\ \%$ using Equation (S41-42), $IQE = k_{\mathrm{et}}p_{\mathrm{m}}/G$ relation, and parameters in Supplementary Table 1, respectively. The dashed line in Supplementary Fig. 13 indicates the result of Equation (S50). If all photons above the band gap of 1.9 eV are absorbed uniformly in the sample of thickness 0.1 μm**,** the AM 1.5 G sunlight illumination corresponds to $10^{22}\ \mathrm{cm}^{-3}\mathrm{s}^{-1}$. The asymptotic decay given by Equation (S50) is visible below the AM 1.5 G sunlight intensity only for the case (b) with $n_{\mathrm{eq}}/10^{3}$. We can see that the internal quantum efficiency depends on the light intensity more strongly as the value of n_{eq} and k_{et} are decreased. Fig. 5b and Supplementary Fig. 11 indicate the importance of reducing the trap states to suppress the decrease of IQE when the semiconductor is less doped, in particular when the carrier extraction rate is small. By increasing the light intensity in the presence of the trap states, the fraction of trapped minority holes increases which leads to the decrease in the fraction of mobile holes. Such imbalance between the majority electron density and the mobile hole density increases with the increase of light intensity. Consequently, the minority holes extraction for oxygen evolution reaction decreases, and competing holes recombination with electrons increases to reduce

the IQE at increased light intensity. Although the IQE can be increased by decreasing the n_{eq} through doping and optimization of particle sizes with large k_{et}, the IQE can be stabilized against the light intensity variation by further decreasing the VB tail states of $Y_2Ti_2O_5S_2$.

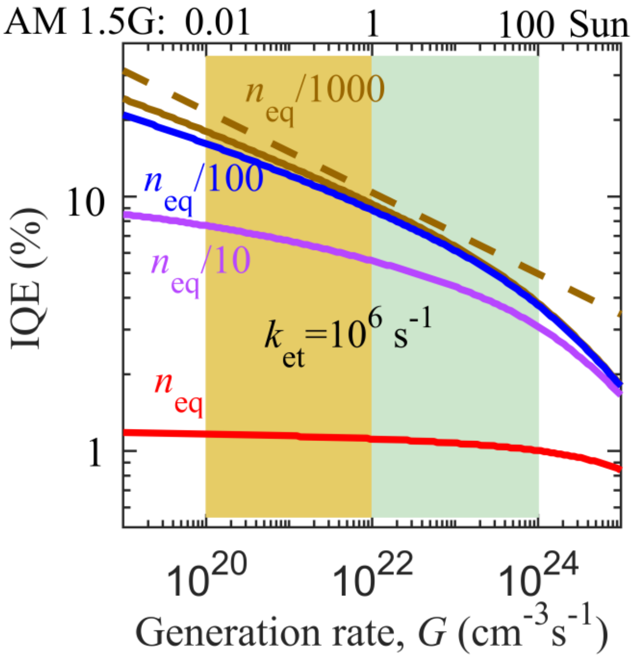

Supplementary Figure 13. Influence of n-type doping density n_{eq} on internal quantum efficiency (IQE) at various charge carrier generation rate G or AM 1.5G light intensity for charge extraction rate $k_{et} = 10^6\ s^{-1}$, in presence of tail trap states. Here, the dotted line represents the solution of analytical IQE model, Equation (S50).

References

1. Maeda, K. & Domen, K. Photocatalytic Water Splitting: Recent Progress and Future Challenges. *J. Phys. Chem. Lett.* **1**, 2655–2661 (2010).

2. Phromma, S., Wutikhun, T., Kasamechonchung, P., Eksangsri, T. & Sapcharoenkun, C. Effect of calcination temperature on photocatalytic activity of synthesized TiO_2 nanoparticles via wet ball milling sol-gel method. *Appl. Sci.* **10**, (2020).

3. Wang, H. et al. Hydrothermal synthesis and tunable multicolor upconversion emission of cubic phase Y_2O_3 nanoparticles. *Adv. Condens. Matter Phys.* **2013**, 347406 (2013).

4. Project, T. M. Materials data on $Y_2Ti_2O_7$ by materials project. (2020) doi:10.17188/1263583.

5. Persson, K. Materials data on $Y_2Ti_2S_2O_5$ (SG:139) by materials project. (2016) doi:10.17188/1200585.

6. Wang, Q. *et al.* Oxysulfide photocatalyst for visible-light-driven overall water splitting. *Nat. Mater.* **18**, 827–832 (2019).

7. McColl, K. & Corà, F. Fast lithium-ion conductivity in the ‘empty-perovskite’ n = 2 Ruddlesden–Popper-type oxysulphide $Y_2Ti_2S_2O_5$. *J. Mater. Chem. A* **9**, 7068–7084 (2021).

8. Nishiwaki, M. & Fujiwara, H. Highly accurate prediction of material optical properties based on density functional theory. *Comput. Mater. Sci.* **172**, 109315 (2020).

9. Cooper, J. K. et al. Physical origins of the transient absorption spectra and dynamics in thin-film semiconductors: The case of $BiVO_4$. *J. Phys. Chem. C* **122**, 20642–20652 (2018).

10. Gardner, R. F. G., Sweett, F. & Tanner, D. W. The electrical properties of alpha ferric oxide—II.: Ferric oxide of high purity. *J. Phys. Chem. Solids* **24**, 1183–1196 (1963).

11. Higashi, T. et al. Transparent Ta_3N_5 photoanodes for efficient oxygen evolution toward the development of tandem cells. *Angew. Chemie Int. Ed.* **58**, 2300–2304 (2019).

12. Nandal, V. et al. Probing fundamental losses in nanostructured Ta_3N_5 photoanodes: design principles for efficient water oxidation. *Energy Environ. Sci.* **14**, 4038-4047 (2021)

13. Iwase, M. et al. One-dimensional anisotropic electronic states in needle-shaped $La_5Ti_2CuS_5O_7$ single crystals grown in molten salt in bridgman furnace. *Cryst. Growth Des.* **19**, 2419–2427 (2019).

14. Wright, A. D. et al. Electron-phonon coupling in hybrid lead halide perovskites. *Nat. Commun.* **7**, (2016).

15. Bernardi, M., Vigil-Fowler, D., Ong, C. S., Neaton, J. B. & Louie, S. G. Ab initio study of hot electrons in GaAs. *Proc. Natl. Acad. Sci.* **112**, 5291–5296 (2015).

16. Karakus, M. et al. Phonon–electron scattering limits free charge mobility in methylammonium lead iodide perovskites. *J. Phys. Chem. Lett.* **6**, 4991–4996 (2015).

17. Monti, M. et al. Efficient intraband hot carrier relaxation in the perovskite semiconductor $Cs_{1-x}Rb_xSnI_3$ mediated by strong electron–phonon coupling. *J. Phys. Chem. C* **122**, 20669–20675 (2018).

18. Rao, A., Wilson, M. W. B., Albert-Seifried, S., Pietro, R. D. & Friend, R. H. Photophysics of pentacene thin films: The role of exciton fission and heating effects. *Phys. Rev. B* **84**, 195411 (2011).

19. Hayes, D. et al. Electronic and nuclear contributions to time-resolved optical and X-ray absorption spectra of hematite and insights into photoelectrochemical performance. *Energy Environ. Sci.* **9**, 3754–3749 (2016).

20. Smolin, S. Y., Choquette, A K., Wang, J., May, S. J. & Baxter, J. B. Distinguishing thermal and electronic effects in ultrafast optical spectroscopy using oxide heterostructures. *J. Phys. Chem. C* **122**, 115–123 (2018).

21. Denis, S. G & Clarke, S. J. Two alternative products from the intercalation of alkali metals into cation-defective Ruddlesden–Popper oxysulfides. *Chem. Commun.* 2356-2357 (2001).

22. Tachiya, M. & Seki, K. Theory of bulk electron-hole recombination in a medium with energetic disorder. *Phys. Rev. B* **82**, 85201 (2010).

23. Olver, F. W. J. et al. *NIST handbook of mathematical functions hardback and CD-ROM*. (Cambridge University Press, 2010).

24. Sze, S. M. *Physics of Semiconductor Devices*. (John Wiley & Sons, 1981).

25. Carslaw, H. S. & Jaeger, J. C. *Conduction of Heat in Solids*. (Clarendon Press, 1986).
